# CORE-Net: A cross-modal orthogonal representation enhancement network for low-altitude multispectral object detection

**DOI:** 10.1371/journal.pone.0340499

**Published:** 2026-04-21

**Authors:** Daoze Tang, Shuyun Tang, Dequan Zheng

**Affiliations:** 1 Harbin University of Commerce, Harbin, China; 2 College of Information and Electrical Engineering, China Agricultural University, Beijing, China; University of Southern California, UNITED STATES OF AMERICA

## Abstract

Object detection in visible light (RGB) images is frequently compromised by low-illumination conditions, whereas infrared (IR) imaging typically exhibits superior robustness in such environments. Multispectral fusion addresses this limitation by leveraging complementary information from both modalities; however, existing methods predominantly rely on intricate fusion modules to integrate cross-modal features, inevitably incurring significant computational overhead and architectural complexity. To mitigate this issue, we propose a novel Cross-modal Orthogonal Representation Enhancement Network (CORE-Net). Diverging from conventional heavy-fusion paradigms, our framework adopts a dual-branch architecture integrated with a streamlined Cross-modal Concatenation Network Framework (CCNF), which achieves efficient feature integration while substantially reducing model complexity. Furthermore, CORE-Net incorporates two distinct components—the Multiple Pooling Convolution Downsampling (MPCD) module and the Refined Integration Network (RINet)—specifically designed to optimize feature extraction capabilities. Extensive evaluations on the DroneVehicle and LLVIP datasets demonstrate that CORE-Net achieves state-of-the-art (SOTA) performance in terms of both detection accuracy and computational efficiency. Ablation studies substantiate the individual and synergistic contributions of each proposed component, while deployment on edge devices further corroborates the model’s practical efficiency. Additionally, qualitative visualizations confirm the model’s efficacy in suppressing background noise and enhancing discriminative fine-grained features. In summary, CORE-Net establishes a robust new paradigm for high-performance and efficient multispectral object detection.

## Introduction

In recent years, optical remote sensing has been widely applied in fields such as autonomous driving, surveillance, environmental monitoring, and urban planning [1–5]. Advances in deep learning (DL) have significantly enhanced object detection accuracy. Two-stage detection algorithms, such as R-CNN [6], Faster R-CNN [7], and SPP-Net [8], rely on region proposal networks to generate candidate bounding boxes for classification and recognition. In contrast, single-stage detection algorithms simultaneously predict both object categories and locations, eliminating the need for region proposal generation. Notable examples include SSD [9], RetinaNet [10], and the YOLO series [11–13]. Remote sensing object detection (RSOD) [14–16] further plays a critical role in target localization and recognition within remote sensing applications.

In previous studies [17,18], most methodologies have been designed and optimized for single-modal target detection using red-green-blue (RGB) and infrared (IR) imagery. However, the performance of these approaches remains limited when detecting targets with subtle or indistinct features [19]. Traditional low-altitude remote sensing target detection predominantly relies on RGB images as the primary data source due to their detailed edge structures, complex textures, and rich color information [20], particularly under optimal lighting conditions. Furthermore, the widespread availability of large-scale RGB datasets provides ample training samples for such tasks. Nevertheless, in extreme environments, RGB images are susceptible to illumination variations, which can lead to detail degradation, while complex backgrounds may result in occlusion or incomplete data coverage [21]. These limitations present significant challenges to enhancing detection accuracy and robustness in low-altitude remote sensing applications. Conversely, infrared images capture thermal radiation patterns and temperature gradients, enabling clear delineation of target contours even under low-light conditions, nighttime operations, long-distance scenarios, or severe occlusion [22,23]. While IR imagery exhibits a distinct advantage over RGB in low-light environments [24,25], it performs suboptimally when target boundaries are ambiguous. Given the complementary nature of RGB and IR modalities in target detection, integrating their synergistic information could substantially improve the performance of multimodal low-altitude remote sensing systems.

Previous research on RGB-infrared (RGB-IR) object detection has faced two primary challenges in complementary feature extraction and image fusion. First, the distinct imaging principles of RGB and IR modalities lead to inherent inconsistencies in their feature representations [26]. Second, objects with analogous visual or thermal signatures often introduce redundancy during fusion [27], which can degrade model performance. Consequently, the effective integration of multimodal complementary features is critical for improving detection accuracy. Existing RGB-IR fusion strategies broadly fall into three categories: concatenation-based, summation-based, and gated control-based methods [28,29]. The concatenation strategy [30] typically fuses RGB and IR features at the channel level, thereby maximizing the preservation of multimodal information. The summation strategy integrates [31,32] features through pixel-wise weighted addition, ensuring the overall consistency between different modalities. In contrast, the gated control strategy [33,34] employs gated units to dynamically adjust the weights of RGB and IR features, enabling adaptive multimodal fusion. Concatenation-based approaches are further categorized as early, intermediate-level, or late fusion. Early fusion integrates RGB and IR data at the pixel level or input channels. Intermediate-level fusion leverages semantic correlations between modalities by extracting features from each and combining them into enriched representations. Late fusion independently processes both modalities and merges their final detection outputs. Although intermediate-level fusion generally yields superior accuracy, its high computational complexity and memory requirements hinder practical deployment. Hence, a paramount objective in multimodal object detection is to navigate the inherent trade-off between computational demands and the robustness of detection performance.

To address these challenges, this study proposes the Cross-Modal Orthogonal Representation Enhancement Network (CORE-Net), an innovative RGB-IR fusion framework tailored for small-target detection in low-altitude remote sensing imagery. By leveraging orthogonal representation learning, CORE-Net enhances discriminative multispectral feature extraction while mitigating cross-modal redundancy—a key challenge stemming from the limited pixel footprint of targets in aerial remote sensing scenarios.

The main contributions of this study are as follows:

- A series of structural optimization modules—including Multiple Pooling Convolution Downsampling (MPCD) and Refined Integration Network (RINet)—is proposed to enhance discriminative feature extraction capabilities in visually complex environments.
- A Cross-modal Concatenation Network Framework (CCNF) is introduced, which replaces computationally intensive fusion operators with streamlined channel-wise concatenation. This design reduces the architectural complexity inherent in traditional cross-modal fusion paradigms while maintaining inter-modal feature compatibility.
- A novel multimodal object detection model, CORE-Net, is developed based on CCNF. This model features a collaborative architecture comprising a dual-branch backbone network and a fusion-guided neck network, significantly improving detection accuracy and model generalizability in challenging scenarios.

The structure of this paper proceeds as follows. A survey of the literature is presented in Sect Related Work, which synthesizes contemporary advances in object detection and multimodal object detection specific to the remote sensing domain. Sect Methods furnishes a comprehensive exposition of the architectural blueprint and implementation specifics of our proposed CORE-Net framework. Sect Results delineates the experimental protocol, benchmark performance against prevailing state-of-the-art methods, and a series of ablation studies that critically evaluate the model’s constituent components. Finally, Sect Discussion and Sect Conclusions critically examines the model’s performance, addresses its limitations, and outlines promising directions for future research.

## Related work

### Remote sensing object detection

Remote sensing object detection [Drone-[35,36], a critical subtask in computer vision, leverages deep learning to classify and localize objects in remote sensing images (RSIs). This task faces challenges such as complex backgrounds, arbitrary object orientations, and small target sizes, which necessitate robust methods to minimize false negatives (missed detections) and false positives (erroneous detections). To address these issues, recent studies have proposed optimization frameworks to improve detection accuracy and robustness.

For instance, Li *et al*. [37] introduced the Lightweight Large Selective Kernel Network (LSKNet), which enhances contextual semantic modeling across object categories by stacking LSK modules. Similarly, Bi *et al*. [38] developed the Local Semantic Enhancement Convolutional Network (LSE-Net). By integrating a context-aware Class Peak Response (CACPR) mechanism, LSE-Net extracts discriminative local features and refines semantic representations, thereby improving recognition performance in aerial scenes. Wu *et al*. [39] proposed CBGS-YOLO, a framework addressing object occlusion in cluttered environments through the Ghost and SPD-Conv modules, which strengthen feature extraction and detection precision. In another approach, Zhang *et al*. [40] incorporated a Bidirectional Feature Pyramid Network (BiFPN) into YOLO, utilizing adaptive feature pooling and fully connected fusion layers to retain spatial information, achieving state-of-the-art performance in benchmark evaluations.

While these studies demonstrate progress, they primarily focus on single-modal data (e.g., RGB images). However, single-modal approaches inherently suffer from limited feature representation due to sensor-specific constraints. In contrast, multimodal data fusion can leverage complementary information from diverse sources (e.g., LiDAR, hyperspectral, SAR), offering richer contextual cues for improved detection.

### Object detection with multimodal data

Object detection models are widely applied in practical scenarios such as remote sensing image classification, autonomous driving, and visual question answering. To enhance detection performance, researchers increasingly leverage multimodal data sources, including synthetic aperture radar (SAR), light detection and ranging (LiDAR), infrared (IR), and multispectral (MS) data [41,42]. By integrating complementary features from diverse modalities, these models improve detection accuracy and system robustness while mitigating the risk of object omission inherent to single-modal approaches.

Multimodal fusion methods are broadly categorized into early fusion, intermediate-level fusion [43], and late fusion, which correspond to pixel-level, feature-level, and decision-level strategies, respectively. For instance, Zhang *et al*. [44] proposed SuperYOLO, a pixel-level fusion framework that integrates auxiliary super-resolution (SR) techniques to enhance multi-scale target feature learning. Fei *et al*. [45] introduced ACDF-YOLO, employing an efficient shuffle attention (ESA) mechanism and a cross-modal difference module (CDM) to optimize global feature extraction while reducing computational redundancy, thereby improving multimodal fusion efficiency. Sharma *et al*. [46] developed YOLOrs, a feature-level fusion-based convolutional neural network, designed for real-time vehicle detection and prediction using multi-scale features. Wang *et al*. [47] proposed YOLOfiv, a dual-stream architecture incorporating an attention mechanism. This model integrates an efficient channel attention (ECA) module and a rotating detection head to enhance accuracy and stability in all-weather remote sensing imagery (ARSI) applications. Building on this, Xie *et al*. [48] addressed spatial misalignment in ARSI detection by incorporating cross-modal local calibration (CLC) and cross-modal global context modeling (CGC) modules.

Existing studies demonstrate the prevalence of multi-branch feature-level fusion in remote sensing applications. However, persistent challenges include modality inconsistency (e.g., spatial or spectral mismatches) and information redundancy (e.g., overlapping features across modalities). To address these challenges, we propose CORE-Net, a dual-branch feature extraction framework. By fusing features from RGB and infrared images at multiple levels, the model enhances the extraction of semantic and spatial features, achieves cross-modal feature complementarity, and improves target detection accuracy and robustness while reducing computational overhead.

## Methods

**Fig 1:**
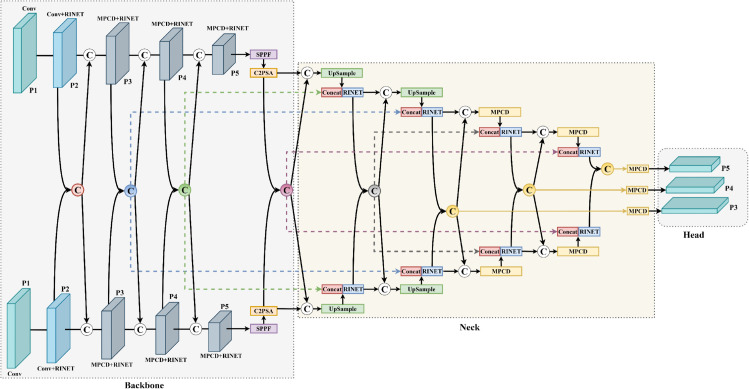
Architectural overview of the CORE-Net model.

To address the challenges of object detection in low-altitude remote sensing imagery under demanding conditions—including small object size, dense spatial distributions, and low-light environments—we introduce the CORE-Net model. This architecture optimizes the cross-modal integration of visible light and infrared spectral features using computationally efficient architectural components. The operational workflow of CORE-Net is detailed in Algorithm 1, while its architecture is illustrated in Fig 1.

**Algorithm 1. Workflow of the CORE-Net model.**



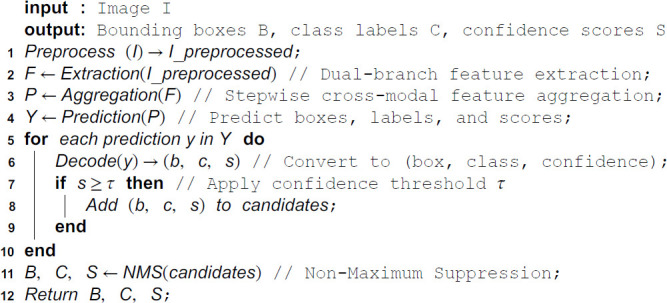



Conventional multimodal (RGB+IR) detection architectures typically employ complex fusion modules that achieve significant performance gains at the expense of substantial computational complexity. The CORE-Net framework introduces a Cross-modal Concatenation Network Framework (CCNF) that establishes multi-spectral feature integration through hierarchical channel concatenation operations. This configuration enables progressive cross-channel feature fusion through cascaded extraction components, forming a symmetrical dual-branch backbone and neck network architecture that maintains computational efficiency while effectively leveraging multimodal information.

The complete CORE-Net system integrates multiple innovative components including MPCD and RINet modules. Its architecture begins with a dual-branch, cross-modal backbone for parallel feature extraction. These features are then progressively fused within the neck network. Subsequently, the consolidated feature representation undergoes final processing in the task head to generate bounding box predictions and classification labels.

### Cross-modal concatenation network framework

**Fig 2:**
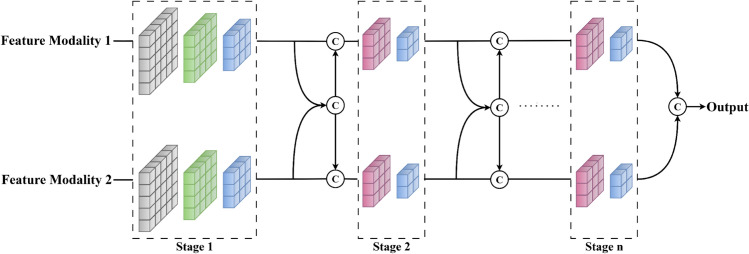
Structure of the CCNF.

The architecture of the Cross-modal Concatenation Network Framework (CCNF) is illustrated in Fig 2. This framework is designed to achieve efficient cross-modal feature fusion. Unlike conventional single-layer fusion approaches, CCNF adopts a multi-stage feature fusion strategy.

The core principle of the proposed framework is the multi-stage fusion of multimodal features. At each stage, features are concatenated along the channel dimension according to a predefined ratio. These fused representations are then propagated to subsequent layers. Although relying on computationally simple operations, this design enables robust performance with minimal computational overhead.

### Multiple pooling convolution downsampling

In remote sensing image processing, challenges such as cluttered backgrounds, significant variations in target sizes, low spatial resolution, and dynamic lighting conditions pose substantial obstacles to effective multi-scale feature extraction. Conventional methods often utilize convolutional (Conv) modules for downsampling. However, standard Conv modules predominantly rely on fixed-scale convolutional kernels, inherently restricting their capacity to capture multi-scale features, consequently compromising detection accuracy for targets of diverse dimensions. Although downsampling techniques facilitate the aggregation of local features while reducing computational complexity and model parameters, they simultaneously constrain the receptive field, which may result in the loss of critical spatial or contextual information.

**Fig 3:**
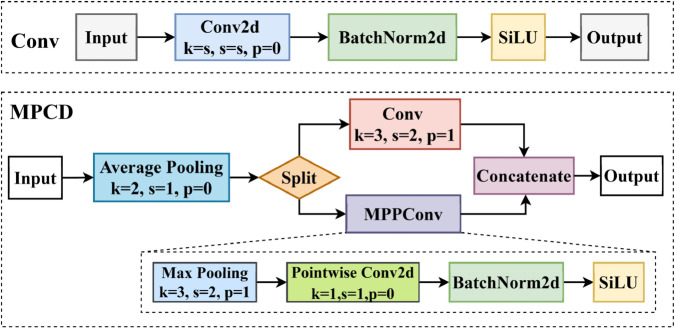
Schematic representation of the MPCD in CORE-Net.

To address the challenge of feature extraction, we propose an adaptive feature fusion module termed Multiple Pooling Convolutional Downsampling (MPCD). The MPCD first applies local average pooling to the input feature map and processes the features through two parallel branches: one branch employs a convolutional (Conv) module, while the other combines max pooling with pointwise convolution. By independently processing features through these branches, the MPCD preserves spatial details captured by the Conv module while incorporating high-frequency information from the max pooling branch. This dual-path architecture enables multi-scale feature representation and reduces computational complexity without sacrificing critical information. A structural comparison with the baseline Conv module is shown in Fig 3.

Eq (1) describes the average pooling operation used in MPCD. Let $X\in R^{C\times H\times W}$ denote the input feature map, where *C*, *H*, and *W* correspond to the number of channels, height, and width, respectively. The value of *X* at spatial position (*i*, *j*) is denoted by *X*(*i*,*j*), and *K* corresponds to the pooling kernel size (scalar or tuple).

$$Y_{\text{avg}}(i,j)=\frac{1}{k^{2}}\sum_{n=0}^{k-1}\sum_{m=0}^{k-1}X(i+n,j+m)$$ (1)

The input feature map is partitioned into two distinct sub-tensors along the channel axis.

The first sub-tensor undergoes a 3×3 convolutional layer (kernel size=3, stride=2, padding=1) to capture spatially localized patterns, as formalized in Eq (2). Here, ${𝐖}_{\text{conv}}$ denotes the convolutional kernel weight matrix, and $b_{\text{conv}}$ is the bias term.

$$Y_{\text{conv}}(i,j)=\sum_{n=0}^{k-1}\sum_{m=0}^{k-1}X_{\text{avg}}(i+n,j+m)\cdot W_{\text{conv}}(n,m)+b_{\text{conv}}$$ (2)

The second component employs Max Pooling Pointwise Convolution (MPPConv) to capture global information. MPPConv applies max pooling to extract the most significant features, thereby expanding the receptive field. It then integrates inter-channel features via pointwise convolution to enhance multi-scale information representation, as defined in Eq (3) and Eq (4).

$$Y_{\text{max}}(i,j)=\max_{n=0}^{k-1}\max_{m=0}^{k-1}X_{\text{avg}}(i+n,j+m)$$ (3)

$$Y_{\text{point}}(i,j)=Y_{\text{max}}(i,j)\cdot W_{\text{point}}(0,0)+b_{\text{point}}$$ (4)

Finally, the two components are concatenated and fused to integrate local and global features effectively.

MPCD integrates both average pooling and maximum pooling operations, combining their advantages to reduce spatial data redundancy while improving computational efficiency. This integration simultaneously enlarges the receptive field and enhances the model’s ability to prioritize salient features within the target region, thereby minimizing the model’s reliance on irrelevant information. Furthermore, the component mitigates overfitting by suppressing excessive background attention during feature extraction, resulting in more robust and generalizable representations.

### Refined integration network

To enhance adaptability to targets of varying scales and orientations in complex backgrounds, this paper proposes a Refined Integration Network (RINet). RINet employs multi-branch architecture to efficiently capture key directional and multi-scale features in remote sensing images. It prioritizes edge-related and high-frequency features while optimizing computational efficiency and feature extraction accuracy.

**Fig 4:**
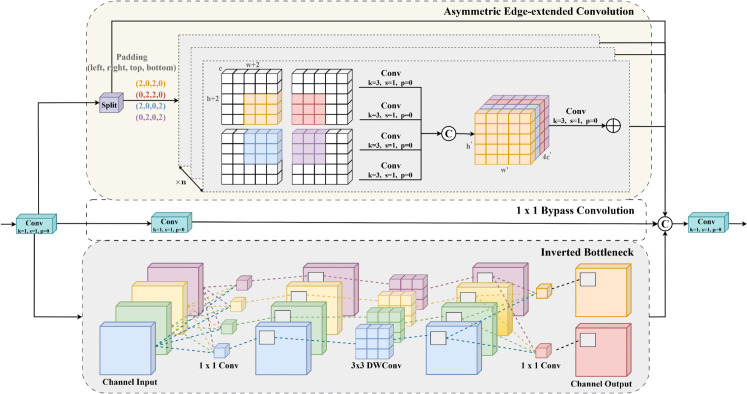
Workflow of the RINet.

As illustrated in Fig 4, RINet comprises three branches:

1. The Asymmetric Edge-extended convolution (AEEC) module branch, designed to strengthen directional feature representation and emphasize edge-specific target features;
2. The 1 × 1 bypass convolutional branch, which redistributes and calibrates channel-wise features;
3. The inverted bottleneck structure branch, composed of a depthwise convolution followed by two 1 × 1 convolutions, to enhance hierarchical multi-scale feature extraction.

In the AEEC module, the input feature map is first replicated four times to generate four parallel intermediate branches. Each branch is padded with two pixels at its four cardinal directions (top-left, top-right, bottom-left, and bottom-right). Subsequently, each branch is independently processed by a 3 × 3 convolutional layer for feature extraction. The outputs of the four branches are then concatenated along the channel dimension, followed by a 3 × 3 convolutional layer with stride 1 to fuse spatial and channel features. This design strengthens the model’s attention to edge-related features, expands the receptive field, and integrates global context, thereby improving the detection of small targets, edge-region objects, and multi-scale objects. Consequently, the module achieves higher detection accuracy in complex backgrounds.

In the inverted bottleneck branch, the input feature map first undergoes channel-wise information fusion via a 1×1 convolutional layer while maintaining the original channel count. The fused features are then processed by a 3×3 depthwise convolutional layer (DWConv) to extract spatial details. Finally, a 1×1 convolutional layer integrates cross-channel information while reducing the channel dimension by half. This design allows the inverted bottleneck branch to supplement feature representations and attention scales for the AEEC module branch, striking a balance between model performance and computational efficiency.

### Spatial pyramid pooling - Fast

**Fig 5:**
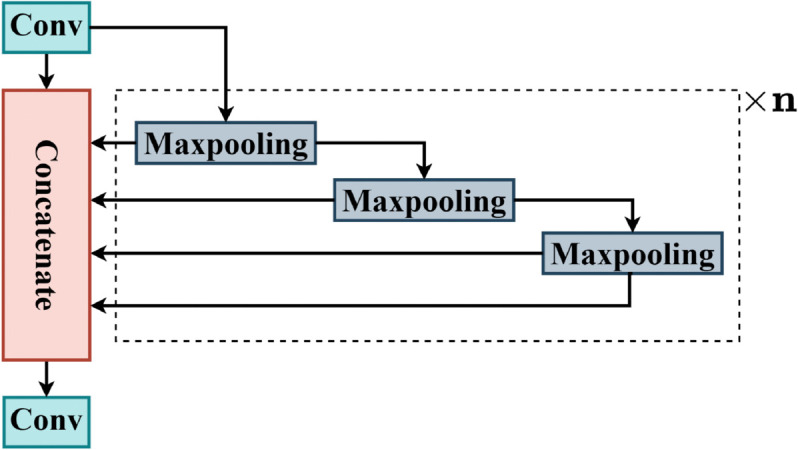
Structure of the SPPF module.

In the CORE-Net model, a Spatial Pyramid Pooling - Fast (SPPF) [49] module is incorporated, as illustrated in Fig 5. This module hierarchically processes feature maps derived from the final backbone network layer by integrating convolutional operations with a series of max-pooling layers.

By default, three consecutive max-pooling layers are employed. Initially, the input feature map is processed through a convolutional layer to integrate cross-channel information and adjust the channel dimensionality. Subsequently, multi-scale salient features across varying receptive fields are extracted via cascaded max-pooling layers. The outputs from each pooling stage are concatenated along the channel dimension to form a comprehensive multi-scale feature representation. Finally, a concluding convolutional layer fuses these aggregated features and refines the channel dimension.

This architecture enhances the model’s sensitivity to salient features, improves the distinction between target characteristics and background noise, and strengthens the integration of multi-scale spatial context. Furthermore, it achieves an optimal balance between computational efficiency and detection performance through hierarchical feature abstraction and parameter optimization.

### Cross-stage-partial convolution with position-sensitive attention

**Fig 6:**
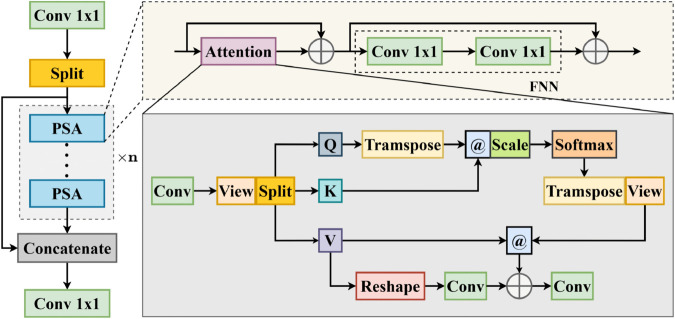
Structure of the C2PSA module.

The CORE-Net model further incorporates the Cross-Stage-Partial Convolution with Position-Sensitive Attention (C2PSA) [50] module, whose architecture is illustrated in Fig 6.

The C2PSA integrates multiple Position-Sensitive Attention (PSA) sub-modules. Each PSA employs a Multi-Head Self-Attention (MHSA) mechanism and a Feed-Forward Neural Network (FNN) comprising two convolutional layers, thereby enhancing the model’s capability to locate and emphasize critical spatial regions. To preserve crucial information, a skip connection is adopted within the component, concatenating the original features with those refined by the PSA. Subsequently, a convolutional operation is applied to the concatenated features to facilitate cross-channel interaction and multi-level feature fusion.

By combining multi-scale convolution with a feature weighting mechanism, the C2PSA module can extract richer and more discriminative feature representations. This design strengthens spatial attention and perceptual capacity in complex environments, effectively addressing the challenge of feature extraction for tiny and occluded objects in complex, low-altitude remote sensing environments.

### Task head

**Fig 7:**
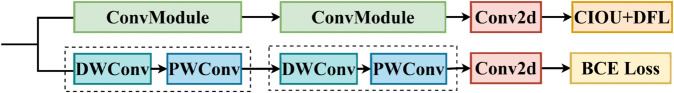
Structure of the task head.

Small objects in low-altitude remote sensing imagery frequently exhibit low contrast relative to the background environment, leading to a tendency toward missed detections in conventional approaches. To address this challenge and enhance the robustness of small object detection, the proposed CORE-Net model employs a task-specific processing head featuring a decoupled dual-branch architecture. As illustrated in Fig 7, this architecture comprises two distinct branches: one dedicated to bounding box prediction and the other to category prediction. Such a decoupled design has been demonstrated in prior studies [50] to enhance detection accuracy while mitigating interference between localization and classification tasks.

The bounding box branch employs two consecutive standard convolutional (Conv) modules for feature extraction from the input feature map. A 1×1 2D convolutional layer is incorporated to strengthen inter-channel feature interactions, while the integration of distributed focal loss (DFL) and complete intersection over union (CIoU) optimizes bounding box localization accuracy.

The classification branch employs two sequential depthwise separable convolutional layers—composed of a depthwise convolution (DWConv) followed by a pointwise convolution (PWConv)—to hierarchically extract discriminative features. These layers are succeeded by a standard 2D convolutional layer coupled with a binary cross-entropy (BCE) loss function to perform category prediction. This parameter-efficient design achieves a favorable trade-off between computational overhead and classification accuracy.

## Results

### Datasets

The DroneVehicle dataset [51], a large-scale visible-infrared aerial benchmark developed by Tianjin University, provides 28,439 spatially aligned image pairs containing 953,087 annotated vehicle instances across five categories (Car, Truck, Bus, Van, and Freight Car). This low-altitude remote sensing dataset is partitioned into three subsets: 17,990 training pairs, 1,469 validation pairs, and 8,980 test pairs, encompassing diverse illumination conditions (daytime, dusk, night) and urban scenarios (roadways, parking facilities, residential zones). All aerial captures were acquired through drone-mounted dual-spectral cameras, with geometric alignment ensured through affine transformations and region cropping during preprocessing.

The LLVIP dataset [52], developed by Beijing University of Posts and Telecommunications researchers, focuses on low-light pedestrian detection with 15,488 registered RGB-IR pairs predominantly captured under nocturnal conditions. This specialized dataset provides 12,025 training pairs and 3,463 test pairs, maintaining temporal-spatial synchronization between modalities to ensure experimental reproducibility.

**Fig 8:**
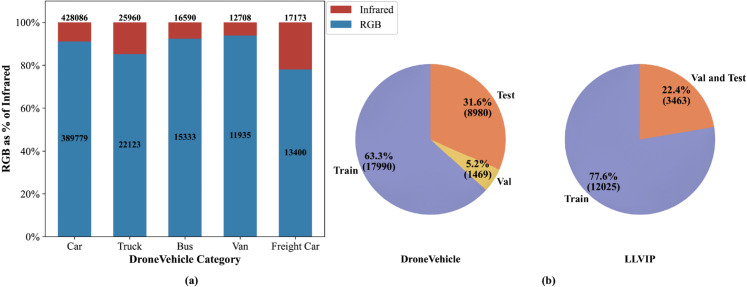
(a) Ratio of RGB to infrared image counts across different categories in the DroneVehicle dataset; (b) image partitioning details of the DroneVehicle and LLVIP datasets.

Experimental evaluations are primarily conducted using the DroneVehicle dataset, while the LLVIP dataset is employed to validate the cross-domain generalization capability of the CORE-Net framework. Fig 8(a) illustrates the category-wise instance distribution for both visible and infrared modalities within the DroneVehicle dataset, whereas Fig 8(b) details the partitioning schemes for these two benchmarks. Notably, the RGB and IR images in these datasets are strictly paired.

### Evaluation metrics

To assess model performance comprehensively, two primary metrics were employed for computational overhead evaluation: parameter count (Params) and floating-point operations (FLOPs). Detection capabilities were quantified using established benchmarks including precision, recall, and Average Precision (AP), with additional analysis through mean Average Precision metrics (${mAP}_{50}$ and ${mAP}_{50-95}$) for multi-threshold evaluation.

The parameter count (Params) quantifies all learnable weights in the model architecture, indicating model size. Floating-point operations (FLOPs) measure the arithmetic computations required per input instance, characterizing computational complexity. Reduced parameter counts correlate with lower memory requirements, while diminished FLOPs suggest greater computational efficiency – particularly advantageous for resource-constrained deployments or real-time inference systems.

$$Precision=\frac{TP}{TP+FP}$$ (5)

$$Recall=\frac{TP}{TP+FN}$$ (6)

Precision and recall are calculated using Eq (5) and Eq (6). Here, True Positive (TP) and True Negative (TN) are the counts of correct positive and negative predictions, respectively; False Positive (FP) is the count of negatives incorrectly predicted as positive; and False Negative (FN) is the count of positives incorrectly predicted as negative.

$$AP=\int_{0}^{1}P(r)\;dr$$ (7)

The Average Precision (AP) for each class, computed as the area under its Precision-Recall (PR) curve, provides a comprehensive summary of detector performance across all confidence thresholds. The calculation method is formally presented in Eq (7).

$$IoU=\frac{TP}{TP+FP+FN}$$ (8)

Intersection over Union (IoU) serves as a standard evaluation metric quantifying the spatial overlap between predicted and ground-truth bounding boxes. This metric exhibits a monotonic relationship with localization accuracy, where higher values indicate greater alignment between predicted and ground-truth regions. The mathematical formulation is formally defined in Eq (8).

$$mAP=\frac{1}{z}\sum_{i=1}^{z}AP_{i}=\frac{1}{z}\int_{0}^{1}P_{i}(r)dr$$ (9)

To address the inherent ambiguity in object boundary delineation, two complementary evaluation metrics are employed: ${mAP}_{50}$ (mean average precision across all categories at a fixed IoU threshold of 0.5) and ${mAP}_{50-95}$ (average precision computed over multiple IoU thresholds from 0.5 to 0.95 with 0.05 increments). These metrics jointly assess model performance in terms of localization consistency and multi-threshold generalization capability. The formal mathematical definitions are provided in Eq (9).

### Experimental environment and hyperparameters

The experimental setup for both the comparative and ablation studies consisted of a server equipped with an AMD EPYC 9654 CPU, 120 GB of RAM, and dual NVIDIA GeForce RTX 4090 GPUs. Detailed hardware and software specifications are provided in Table 1.

**Table 1:** Server-side computing environment specifications.

Parameters	Configuration
CPU	AMD EPYC 9654
Memory	120GB
GPU	NVIDIA RTX 4090 (24GB) × 2
Ubuntu	22.04
Cuda	12.4
Python	3.12.3
Pytorch	2.5.1+cu124
Ultralytics	8.3.70

The experiments on edge device deployment were conducted using an NVIDIA Jetson AGX Orin. Its core components comprise a 12-core Arm^®^ Cortex^®^-A78AE CPU, 32 GB of memory, and a GPU implementing the NVIDIA Ampere architecture. Full specifications are delineated in Table 2.

**Table 2:** Edge computing environment specifications.

Parameters	Configuration
CPU	12-core Arm^®^ Cortex^®^-A78AE v8.2 64-bit CPU
Memory	32GB
GPU	2048-core NVIDIA Ampere architecture GPU with 64 Tensor Cores
Ubuntu	20.04
Jetpack	5.1.3
Cuda	11.4.315
Python	3.8.10
Pytorch	2.1.0a0+41361538.nv23.6
Ultralytics	8.3.70

Training hyperparameters are summarized in Table 3. To ensure experimental consistency, all trials retained identical hardware, software configurations, and hyperparameter settings.

**Table 3:** Hyperparameter specifications for training protocol.

Parameters	Setup
Image Size	640 × 640
Epochs	200
Optimizer	SGD
Batch Size	16
Workers	128
Weight Decay	0.0005
Learning Rate	0.01
Momentum	0.937
Close Mosaic	0
Patience	0

### Comparative experiments

A comprehensive comparative evaluation of performance was conducted on the DroneVehicle dataset, involving the proposed CORE-Net model and a series of baseline models including the LF-MDet [53], C^2^Former-S^2^ANet [54], DDCI-S^2^ANet [55], MDA [56], CM-YOLO-m [57], and UDDet [58].

As presented in Table 4, the proposed CORE-Net demonstrates superior performance across all evaluation metrics. Specifically, compared to LF-MDet, CORE-Net achieves accuracy improvements of 13.6% and 17.7% on the two respective metrics, while simultaneously reducing the parameter count by 95.3% and computational cost by 93.2%. Furthermore, the efficiency advantage of CORE-Net is even more pronounced when benchmarked against heavy-weight models. For instance, relative to UDDet, CORE-Net secures accuracy gains of 4.6% and 9.2%, yet requires only 3.1% of the parameters and 2.8% of the computational resources.

**Table 4:** Performance comparison between CORE-Net and baseline methods on the DroneVehicle dataset.

Method	Input	Params (M)	FLOPs(G)	${mAP}_{50}$(%)	${mAP}_{50-95}$ (%)
LF-MDet [53]	RGB+IR	38.7	77.7	71.8	51.3
C^2^Former-S^2^ANet [54]	RGB+IR	132.5	100.9	74.2	-
DDCI-S^2^ANet [55]	RGB+IR	121.7	124.9	75.7	-
MDA [56]	RGB+IR	72.4	103.7	76.9	-
CM-YOLO-m [57]	RGB+IR	24.1	54.7	79.5	-
UDDet [58]	RGB+IR	57.7	191.9	78.0	55.3
**CORE-Net**	**RGB+IR**	**1.8**	**5.3**	**81.6**	**60.4**

To further assess the adaptability and generalizability of the proposed CORE-Net model, additional robustness verification experiments were conducted on the LLVIP dataset. Baseline models selected for comparison included the ACDF-YOLO [45], YOLOXCPCF [59], FS-Diff [60], RTMF-Net [61], UIRGBfuse [62], DIVFusion [63], FQDNet_n [64], Diff-IF [65], and the proposed CORE-Net model were evaluated.

As illustrated in Table 5, CORE-Net exhibits superior performance on the LLVIP dataset, consistent with its results on the DroneVehicle dataset, effectively outperforming all baseline models. Specifically, compared with RTMF-Net, which possesses a comparable model scale, CORE-Net achieves improvements of 0.7% and 6.5% in two accuracy metrics, respectively, while simultaneously reducing parameter count by 57.1% and computational cost by 54.3%. Notably, despite utilizing only 3.1% of the parameters and less than 0.01% of the computational resources required by the computationally intensive FS-Diff model, CORE-Net surpasses it by 3.3% and 4.6% in the respective accuracy metrics.

**Table 5:** Performance comparison between CORE-Net and baseline methods on the LLVIP dataset.

Method	Input	Params (M)	FLOPs(G)	${mAP}_{50}$(%)	${mAP}_{50-95}$ (%)
ACDF-YOLO [45]	RGB+IR	8.0	12.7	95.9	58.9
YOLOXCPCF [59]	RGB+IR	14.6	15.1	96.4	65.0
FS-Diff [60]	RGB+IR	58.5	64391.6	93.3	62.4
RTMF-Net [61]	RGB+IR	4.2	11.6	95.7	61.3
UIRGBfuse [62]	RGB+IR	4.7	1269.0	87.6	65.3
DIVFusion [63]	RGB+IR	4.4	14454.9	89.8	52.0
FQDNet_n [64]	RGB+IR	4.7	16.9	95.5	61.3
Diff-IF [65]	RGB+IR	23.7	637.7	93.3	59.5
**CORE-Net**	**RGB+IR**	**1.8**	**5.3**	**96.4**	**65.3**

The experimental findings demonstrate that CORE-Net’s design successfully reconciles high accuracy with low computational demands, resulting in a net gain in overall performance.

### Ablation experiments

In this ablation study, YOLO11n serves as the baseline model. Each component and design of the proposed CORE-Net was progressively integrated into this baseline architecture, with the corresponding performance metrics on the DroneVehicle dataset reported in Table 6.

**Table 6:** Component ablation study for CORE-Net on the DroneVehicle dataset.

Method	MPCD	RINet	CCNF	Input	Params (M)	FLOPs(G)	${mAP}_{50}$ (%)	${mAP}_{50-95}$ (%)
Baseline	-	-	-	RGB	2.6	6.3	74.1	51.9
-	✓	-	-	RGB	2.1	5.3	75.1	52.5
-	-	✓	-	RGB	3.3	10.8	76.6	54.1
-	-	-	✓	RGB+IR	2.4	4.7	78.2	57.4
-	✓	✓	-	RGB	2.9	9.8	77.0	54.7
-	✓	-	✓	RGB+IR	1.5	3.0	79.6	58.2
-	-	✓	✓	RGB+IR	2.8	7.0	80.0	59.4
**CORE-Net**	✓	✓	✓	RGB+IR	**1.8(↓30.8%)**	**5.3(↓15.9%)**	**81.6(↑10.1%)**	**60.4(↑16.4%)**

Initially, we evaluated the individual contributions of the three core components: MPCD, RINet, and CCNF. When the MPCD module is implemented independently, accuracy metrics improve by 1.3% and 1.2%, while the parameter count and computational cost decrease by 19.2% and 15.9%, respectively. Notably, MPCD achieves the most significant reduction in parameters among the three components. The isolated implementation of the RINet module yields performance gains of 3.4% and 4.2%; however, this comes at the cost of a moderate increase in model complexity. Conversely, implementing CCNF alone results in accuracy improvements of 5.5% and 10.6%, accompanied by a 7.7% reduction in parameters and a 34.0% decrease in FLOPs. This represents the most substantial improvement in comprehensive performance, primarily attributed to the effective fusion and utilization of multi-modal features. These experiments substantiate the individual effectiveness of CORE-Net’s core components.

Subsequently, we conducted experiments involving pairwise combinations and the complete integration of MPCD, RINet, and CCNF. Across all combination settings, pairwise integrations consistently outperform the individual implementations of their respective components in terms of accuracy. Moreover, the simultaneous implementation of all components achieves superior accuracy compared to any partial integration configuration. This phenomenon demonstrates the synergistic efficacy and mutual reinforcement among the core components of CORE-Net.

In summary, this systematic evaluation validates both the independent validity and the complementary interaction of the CORE-Net architectural elements.

### Edge device deployment experiments

The deployment experiment on edge devices evaluated the proposed CORE-Net against the YOLO11 series baseline models (recognized for real-time performance) using the DroneVehicle dataset.

Experimental results summarized in Table 7 indicate that CORE-Net achieves a 28.1% reduction in computational latency compared to the Baseline-m variant while simultaneously improving accuracy metrics by 2.4% and 3.8%. When compared to the highest-accuracy Baseline-x model, CORE-Net further reduces computational latency by 68.6% with additional accuracy gains of 2.5% and 3.2%. These findings demonstrate that CORE-Net effectively balances accuracy and computational efficiency, showing stronger suitability for high-precision real-time tasks on resource-constrained edge devices.

**Table 7:** Performance comparison between CORE-Net and baseline methods on the DroneVehicle dataset under edge deployment constraints.

Method	Params (M)	FLOPs (G)	${mAP}_{50}$ (%)	${mAP}_{50-95}$ (%)	Edge Latency (s)	Server Latency (s)
Baseline-n	2.6	6.3	74.1	51.9	0.00428	0.00043
Baseline-s	9.4	21.3	78.0	55.8	0.00837	0.00102
Baseline-m	20.0	67.7	79.7	58.2	0.01927	0.00255
Baseline-l	25.3	86.6	79.4	58.3	0.02485	0.00317
Baseline-x	56.8	194.4	79.6	58.5	0.04413	0.00638
**CORE-Net**	**1.8**	**5.3**	**81.6**	**60.4**	**0.01386**	**0.00140**

### Results visualization

Experimental results are comprehensively presented via Precision-Recall (PR) curves, confusion matrices, feature heatmaps, and qualitative visualizations to demonstrate the overall performance superiority of CORE-Net over the baseline model. To ensure a fair and consistent comparison, we adhere to the baseline settings established in the ablation study, thereby explicitly attributing the performance gains to the novel architectural components and structural innovations of CORE-Net.

The PR curve serves as a pivotal metric for evaluating model performance, particularly in scenarios characterized by class imbalance. It elucidates the trade-off between precision and recall across varying decision thresholds, facilitating both intuitive interpretation and rigorous quantitative analysis. Typically, precision exhibits a downward trend as recall increases; lowering the classification threshold captures more True Positives (TP) but inevitably introduces additional False Positives (FP). A superior model demonstrates a marginal decay in precision as recall increases, whereas a suboptimal model suffers a precipitous decline, indicating limited discriminative capability between positive and negative samples. Consequently, a curve approaching the top-right corner—corresponding to a larger Area Under the Curve (AUC)—signifies superior overall performance and an effective equilibrium between precision and recall.

**Fig 9:**
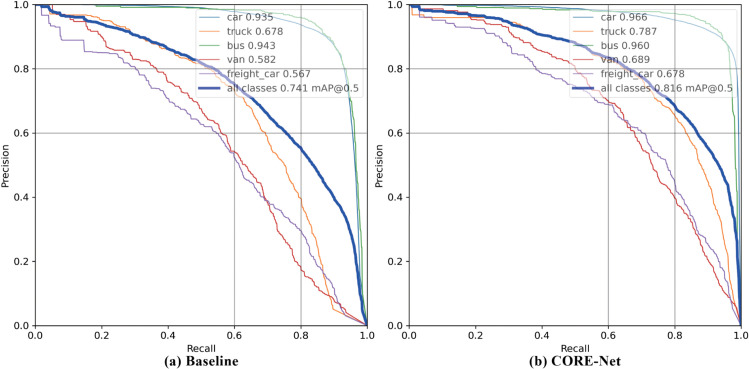
Precision-recall curves for CORE-Net and baseline methods on the DroneVehicle dataset.

Fig 9 illustrates the comparative PR curves for (a) the baseline model and (b) the proposed CORE-Net. With the aid of auxiliary reference lines, it is observed that the all-category PR curve of the baseline intersects the function *y* = *x* approximately at the point (0.70, 0.70), whereas the CORE-Net curve intersects at (0.75, 0.75). This shift demonstrates that the CORE-Net curve consistently approaches the ideal top-right corner and yields a higher AUC value. This indicates that CORE-Net maintains high precision even as recall improves, displaying a superior capability to distinguish true positives from background noise while effectively suppressing false positives. These results confirm that CORE-Net achieves a more optimal precision-recall trade-off, thereby outperforming the baseline model in overall detection performance.

The confusion matrix serves as a foundational evaluation tool for assessing classification model performance. This tabular visualization presents predicted and actual class labels in parallel alignment, conventionally with columns denoting true classes and rows indicating predicted classes (or inversely arranged). Each cell contains the count of samples corresponding to a unique actual-predicted class pair. Diagonal entries correspond to correct classifications, whereas off-diagonal elements quantify classification errors.

**Fig 10:**
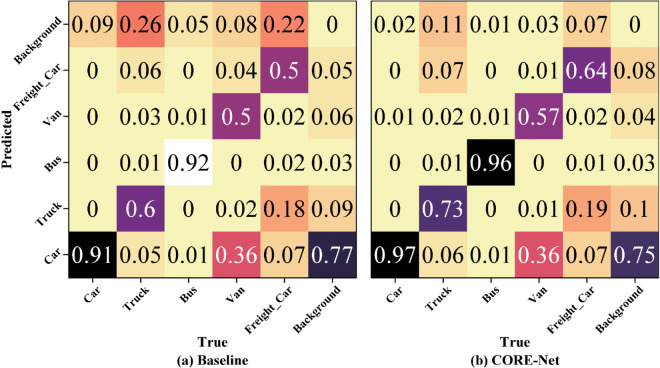
Confusion matrices for CORE-Net and baseline methods on the DroneVehicle dataset.

Fig 10 displays normalized confusion matrices for the (a) baseline and (b) CORE-Net models. Each matrix column is normalized to visualize prediction distributions across categories while mitigating class imbalance bias. Diagonal elements (top-right to bottom-left) denote correct classifications (true positives/negatives), with off-diagonal elements representing misclassifications. Specifically, the top row of off-diagonal elements indicates false negatives where true positives are misclassified as background, while the rightmost column corresponds to false positives where background regions are erroneously classified as target categories. Remaining off-diagonal elements reflect inter-class confusion errors. The CORE-Net matrix exhibits intensified diagonal dominance and suppressed off-diagonal values compared to the baseline, demonstrating enhanced classification performance through reduced error propagation.

Grad-CAM [66] is utilized to produce visual heatmaps, facilitating a comparative analysis of feature attention patterns across the evaluated models. The resulting heatmaps illustrate the regions of interest upon which the model focuses during object detection, with gradations of color intensity indicating the degree of attention allocated to different areas. By comparing these heatmaps against the ground-truth target regions, it is possible to evaluate whether the model effectively directs its attention to the relevant objects in the image.

**Fig 11:**
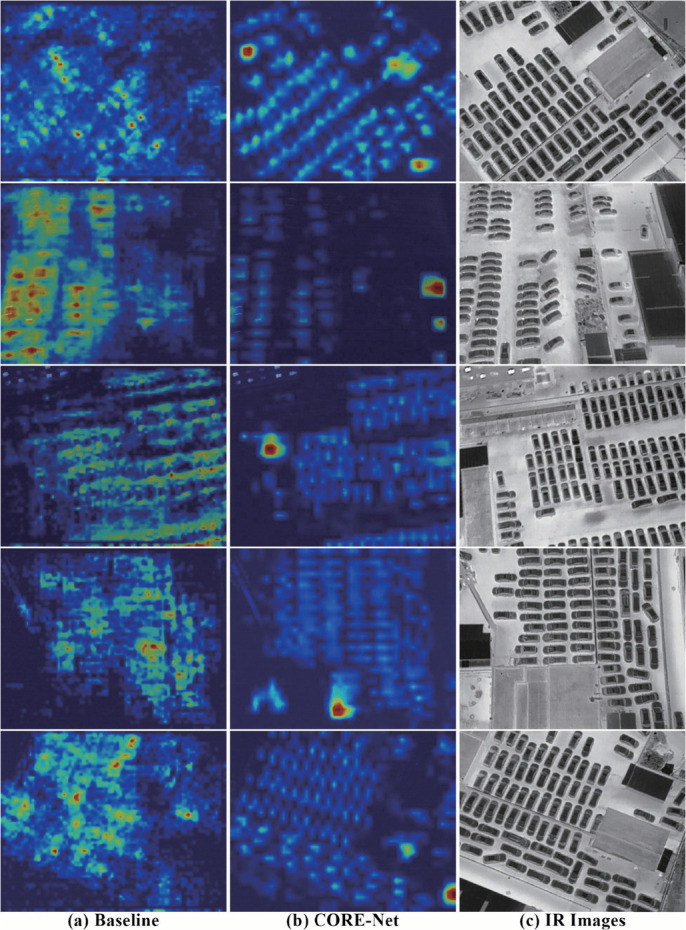
Heatmap comparison on DroneVehicle dataset samples: (a) baseline method, (B) CORE-Net, and (C) corresponding infrared frames.

Fig 11 presents a comparative visualization of several randomly selected samples from the DroneVehicle dataset, illustrating: (a) feature activations of the baseline model, (b) salient regions identified by the proposed CORE-Net, and (c) corresponding infrared frames that serve as visual references for target feature comparison. The baseline model exhibits more pronounced attention toward target regions; however, it fails to adequately differentiate between densely distributed targets, leading to suboptimal generalization in relevant scenarios and resulting in target confusion or missed detections. In contrast, for clusters of small and dense targets, the feature activations generated by CORE-Net demonstrate finer granularity and more clearly delineated region boundaries. It is noteworthy, however, that despite CORE-Net’s effective feature discrimination, residual background activations persist, which may cause certain areas to be erroneously overemphasized. This issue may stem from feature similarities between annotated targets and environmental elements.

**Fig 12:**
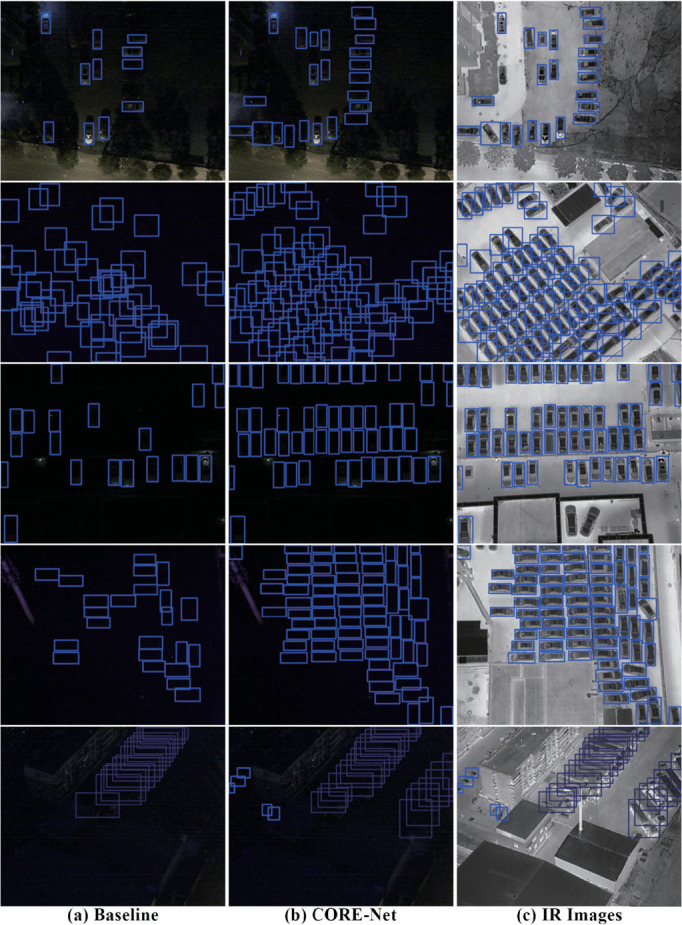
Detection result comparison on DroneVehicle dataset samples: (a) baseline method, (B) CORE-Net, and (C) infrared frames overlaid with CORE-Net detections.

A subset of samples from the DroneVehicle dataset was randomly selected for visual comparison. As illustrated in Fig 12, the baseline model predictions (a), CORE-Net predictions (b), and infrared reference frames overlaid with CORE-Net detections (c) are systematically presented. Comparative analysis reveals that CORE-Net demonstrates enhanced robustness against ambient lighting variations and cluttered backgrounds. Specifically, the proposed model achieves superior detection accuracy for distant small targets, low-light objects, and densely clustered or occluded instances compared to the baseline, exhibiting higher correct detection rates in these challenging scenarios. These improvements can be attributed to the algorithm’s effective fusion of multi-spectral features.

## Discussion

The experimental results demonstrate that the proposed CORE-Net model effectively balances high detection accuracy with low computational cost for low-altitude multispectral object detection. By leveraging a dual-branch architecture and a streamlined Cross-modal Concatenation Network Framework (CCNF), which uses simple channel concatenation instead of complex fusion modules to efficiently integrate RGB and IR features.

Ablation studies confirm the individual and synergistic contributions of the core components. The MPCD module significantly enhances multi-scale feature representation and reduces spatial redundancy, leading to a notable decrease in model parameters. The RINet strengthens the model’s capacity to discern targets across varying scales and orientations, particularly in cluttered backgrounds. The CCNF itself was instrumental in achieving substantial gains in accuracy with reduced FLOPs, demonstrating that efficient cross-modal integration can be realized without resorting to complex gating mechanisms. The collective integration of these components within CORE-Net yields superior performance on both the DroneVehicle and LLVIP benchmarks, underscoring the model’s robustness and generalizability across different low-altitude sensing tasks.

Qualitative analyses, including Grad-CAM visualizations and detection result comparisons, provide further evidence of the model’s advanced capabilities. CORE-Net focuses more precisely on small and dense objects than baselines, reducing missed detections in challenging conditions like low light and occlusion. Nevertheless, the visualizations also reveal a limitation: the model occasionally allocates non-trivial attention to non-salient background regions. This phenomenon indicates a potential avenue for future refinement, wherein feature prioritization mechanisms could be further optimized to suppress irrelevant contextual information without compromising the detection of genuine targets.

## Conclusions

This paper presents CORE-Net, a novel network for efficient and accurate RGB-IR object detection in low-altitude remote sensing. The model successfully addresses key challenges in multispectral fusion through a dual-branch architecture and a simple yet effective Cross-modal Concatenation Network Framework (CCNF).

Supported by the MPCD and RINet modules, CORE-Net achieves superior detection performance on standard benchmarks while maintaining significantly lower computational complexity than existing methods. The model’s efficiency is further validated through deployment experiments on edge devices, confirming its suitability for real-time, resource-constrained applications.

In conclusion, CORE-Net establishes a streamlined and powerful paradigm for multispectral object detection, effectively balancing high precision with low computational overhead. Future work will focus on augmenting the model’s feature discrimination capabilities to reduce background interference and exploring self-supervised learning strategies to leverage unlabeled multispectral data for further performance enhancement.

## Supporting information

S1 File The DroneVehicle and LLVIP datasets are publicly available via https://github.com/VisDrone/DroneVehicle and https://github.com/bupt-ai-cz/LLVIP, respectively. (DOCX)

S2 File The CORE-Net implementation and source code are accessible at https://github.com/DaozeTang/CORE-Net. (DOCX)
