# Supplementary material for: CORE-Net: A cross-modal orthogonal representation enhancement network for low-altitude multispectral object detection
Source: PLoS One. 2026 Apr 21;21(4):e0340499. doi: 10.1371/journal.pone.0340499 (PMC13099095; doi:10.1371/journal.pone.0340499)
Supplement: S1 File — (DOCX) [file pone.0340499.s001.docx]

S1 File. The DroneVehicle and LLVIP datasets are publicly available via https://github.com/VisDrone/DroneVehicle and https://github.com/bupt-ai-cz/LLVIP, respectively.
