# Supplementary material for: CORE-Net: A cross-modal orthogonal representation enhancement network for low-altitude multispectral object detection
Source: PLoS One. 2026 Apr 21;21(4):e0340499. doi: 10.1371/journal.pone.0340499 (PMC13099095; doi:10.1371/journal.pone.0340499)
Supplement: S2 File — (DOCX) [file pone.0340499.s002.docx]

S2 File. The CORE-Net implementation and source code are accessible at https://github.com/DaozeTang/CORE-Net.
